# Advancing proficiency testing for ultra in resource-limited settings using dried tube specimen: A study by SRL-Uganda

**DOI:** 10.1371/journal.pone.0282650

**Published:** 2023-03-10

**Authors:** Joel Kabugo, Joanita Namutebi, Isa Adam, Dennis Mujuni, Didas Tugumisirize, Oola Denis, George William Kasule, Ivan Ibanda, Beatrice Orena, Henry Byabajungu, Elizabeth Nampewo, Moses Joloba

**Affiliations:** 1 Uganda WHO Supranational Reference Laboratory (SRL), Kampala, Uganda; 2 GENLAB Solutions International Limited, Kampala, Uganda; 3 Uganda National TB and Leprosy Program/Ministry of Health (MoH/NTLP), Kampala, Uganda; 4 Central Public Health Laboratories (CPHL), Kampala, Uganda; 5 Makerere University, College of Health Sciences, Kampala, Uganda; Southwest Jiaotong University, CHINA

## Abstract

**Background:**

Proficiency testing (PT) has been hard to set up due to cost limitations and technical capacity. Conventional Xpert MTB/RIF PT programs use liquid and culture spots which require stringent storage and transportation conditions with cross-contamination chances prevalent. These setbacks prompted the use of dried tube specimens (DTS) for Ultra assay PT. For continuity of PT provision, stability of DTS and compatibility with testing protocols when kept for a long period needs to be established.

**Methods:**

DTS were prepared from known isolates inactivated using a hot air oven at 85°C. 100μl of bacterial suspensions were aliquoted and dried inside a Biosafety cabinet. Panel validation was done to establish the baseline Deoxyribonucleic acid (DNA) concentration in terms of cycle threshold (Ct) value. DTS aliquots were shipped to participants to test and report within six weeks. The remaining DTS were kept at 2–8°C and room temperature for one year with testing at six months. Twenty (20) DTS samples per set remaining at one year were heated at 55°C for two weeks before testing. The means of the different samples were compared to validation data using paired t-tests. Boxplots were designed to visualize the differences in the medians of the DTS.

**Results:**

Overall mean Ct value increased by 4.4 from the validation to testing after one year at the different storage conditions. Samples heated at 55°C showed a 6.4 Ct difference from validation data. Testing done at six months on 2–8°C stored items showed no statistical difference. At all the remaining testing times and conditions, P-values were less than 0.008 although the absolute mean Ct when compared showed slight increments and accommodated differences for the detection of *MTB* and rifampicin resistance. Median values for samples stored at 2–8°C were lower compared to those at room temperature.

**Conclusion:**

DTS stored at 2–8°C remain more stable for one year compared to higher temperatures and can be consistently used as PT materials in more than one PT round for biannual PT providers.

## Introduction

Bacteriologically confirming methods for *Mycobacterium tuberculosis (MTB)* have extensively evolved from those focusing only on bacilli morphology to those performing genomic analysis of the bacilli. The Xpert MTB/RIF assay (Cepheid, Sunnyvale, California, United States) and its successor the Ultra assay are the most widely used molecular point of care (POC) tests for the detection of MTB and rifampicin resistance [1,2]. The suboptimal sensitivity of Xpert MTB/RIF in *MTB* smear-negative specimens in HIV-positive people led to modifications of the assay resulting in a more sensitive test recommended by the WHO in 2018 [3]. Ultra was rolled out in Uganda in 2018 and by end of 2021 over 255 machines were running on the Ultra platform for (*MTB*) testing [4].

To ensure the accuracy and consistency of Ultra testing, external quality assurance needs to be performed regularly via routine proficiency testing (PT). A PT mechanism can independently check conformance of the pre-analytic, analytic, and post-analytic phases of analysis in laboratory quality management systems and identify areas that require improvement [5,6]. Various methods have been innovated to prepare and distribute Ultra PT and these include; lyophilized samples, liquid, dried culture spot and dried tube specimen (DTS) which have been mainly implemented in well-resourced countries [6,7]. In many lower-middle-income countries (LMIC), PT programs have been hard to set up due to costs and limitations in technical capacity. In Uganda, the national tuberculosis reference laboratory (NTRL) which is also a WHO Supra-National Reference Laboratory (Uganda-SRL) initiated the preparation of Ultra panels using a liquid matrix which was associated with the need for special storage and transportation conditions, poor stability and high rates of cross-contamination during preparation stages [8,9]. Moreover, effective PT should be conducted biannually requiring preparing of fresh panels for each round which is costly and labor-intensive [8].

To overcome the rigorous and expensive biannual preparations processes while maintaining semiannual PT rounds, the Uganda-SRL adopted the use of DTS, a method that had been used by the United States centers for disease control and prevention (CDC) and in other disease PT schemes such as HIV viral load testing [8,10,11]. We designed this study to demonstrate the stability of DTS panels and compatibility with testing protocol when stored under different environmental conditions for one year.

## Materials and methods

This was a longitudinal study initiated in November 2019 and completed in June 2021 with all activities conducted at the Uganda-SRL. Activities performed included Ultra panel preparation, validation, packaging, distribution and storage of known PT materials of *MTB* and Non-Tuberculosis Mycobacterium (NTM) panels in form of DTS.

### Laboratory methods

Panels were prepared through a series of steps from culturing of colonies to heat inactivation of the harvested bacilli. Eight Stored isolates with known genomic and phenotypic results obtained from the WHO-institute of tropical medicine (ITM)-Antwerp (https://www.itg.be/E/administrator-of-the-worlds-largest-collection-of-tbcstrains) for routine quality control of laboratory processes were used for this study. The production of the DTS followed some of the steps that have been described by Kyle et al 2020 [12]. Briefly, MTB isolates were grown on MGIT with confirmation of MTB-complex (MTBC) done Using SD bioline test (catalog number and description: 08FK50 TB Ag MPT64 Rapid) and Zeihl Neelsen as described in SRL standard operating procedures (SOPs) “S1 File” and WHO guidelines [13,14].

All grown MTB isolates were inactivated at 85°C in the hot air oven for 60 minutes. To verify the expected results and set desired sample dilution factor, 2.5mls of each inactivated stock was pipetted off and a 1:10 dilution was made with the addition of 10μl of blue food color added for visibility before drying the DTS inside the biosafety cabinet for seven days. Using proof of inactivation and pretest results, five stocks with the lowest cycle threshold (Ct) value means and standard deviation of ≤ 3 were selected to comprise the PT panel as shown in Table 1. A standard deviation of ≤ 3 was selected due to consistency in homogeneity obtained with it during previous PT panel preparations [12]. Samples selected included: two *MTB*-detected rifampicin-sensitive, two *MTB*-detected rifampicin-resistant and one NTM as the negative sample.

**Table 1:** Pretest results obtained with a 1:10 serial dilution.

Sample stock	*MTB* detection	rifampicin resistance	rpoB1 mean Ct	rpoB1 SD Ct
2019/2/S-1	Low	Detected	24.82	0.83
2019/2/S-2	Low	Detected	24.88	1.85
2019/2/S-3	Low	Detected	23.52	0.94
2019/2/S-4	Low	Not detected	22.84	1.23
2019/2/S-5	Low	Not detected	25.3	1.04
2019/2/S-6	Low	Not detected	23.72	0.97
2019/2/S-7	Not detected	Not applicable	0	0
2019/2/S-8	Not detected	Not applicable	0	0

From Table 1 above, stock samples (2019/2/S-1, 2019/2/S-3, 2019/2/S-7, 2019/2/S-4 and 2019/2/S-6) were selected and renamed as DTS 2019 A-1, DTS 2019 A-2, DTS 2019 A-3, DTS 2019 A-4 and DTS 2019 A-5 respectively. Serial dilutions of 1:3 (sample: distilled water) were prepared from the stocks. For each food-colored sample solution stock, 4ml capacity cryo-tubes were labeled and aliquoted with100μl of the homogenized final dilution. The 1:3 serial dilution was selected to give an increased *MTB* DNA concentration in the samples and obtain a medium-range reading on the Ultra as the MTB DNA concentration range for this study.

All the prepared DTS were then dried with caps off inside a functional biosafety cabinet (BSC-II) for seven days to improve drying and ensure an aseptic environment. A validation run was performed after the DTS had dried by selecting twenty (20) tubes randomly for each panel set and running them on Ultra assay independently by different trained and competent personnel at Uganda-SRL immediately. The validation results were used as a standard of reference to the results obtained from subsequent testing at the different study conditions.

### Proficiency testing programme

After validation, PT panels were shipped at room temperature to two hundred and thirty-five (235) testing sites with instructions to report results within six weeks after the date of dispatch. Among the participants sent, twenty-five (25) participants were outside Uganda while two hundred ten sites (210) were within Uganda. All the participating sites out of Uganda were national reference laboratories (NRLs) while sites in Uganda included regional referral hospitals 8.1% (17/210), general hospitals 57.6%(121/210), health center four 24.3%(51/210) and health center three 10%(21/210) level sites representative of the TB diagnostic network. Each site had to record both the quantitative and qualitative results i.e., *MTB* and rifampicin detection and the Ct values using a standardized form shipped together with the DTS. All participants in the PT scheme were required to sign a confidentiality waiver to allow Uganda-SRL as a PT provider to use all the information obtained from the participant.

The remaining panel sets were portioned for storage at 2–8°C inside a refrigerator and room temperature for further testing at six (6) months and one year. A total of four hundred (400) DTS were tested for all the set conditions (at six months, one year and after two weeks of incubation in a hot air oven). Two hundred forty (240) were stored at room temperature and 160 at 2–8°C. At each interval of testing, twenty (20) tubes per DTS 2019 A-1, DTS 2019 A-2, DTS 2019 A-4 and DTS 2019 A-5 were tested on the storage conditions listed above. After one year, 20 DTS sample IDs i.e., DTS 2019 A-1, DTS 2019 A-2, DTS 2019 A-4 and DTS 2019 A-5 were randomly picked from a set stored at room temperature and then incubated further at 55°C using a hot air oven for two weeks before testing on Ultra. The negative sample DTS 2019 A-3 was excluded after six weeks of testing since no DNA was present for testing under these different storage conditions.

All results were entered after each testing interval into a customized Microsoft excel sheet that was used to compile, clean and analyze the data generated as shown in “S2 File”. Ct values of rpoB1 were used as a reference to determine whether there is deterioration in deoxyribonucleic acid (DNA) in *MTB* DTS stored at different conditions.

### Statistical methods

Data was analyzed in STATA v16. Variables were summarized into medians (IQR) and means (SD). The means of the different samples were compared to the day “0” means (Validation), using paired t-test [15,16]. To avoid the inflation of alpha, this was adjusted by the Bonferroni method and the means were statistically different if they had a p-value <0.008. Boxplots were contrived to identify any differences in the cycle threshold median values of the different samples at their respective storage conditions over time.

### Ethical consideration

This study did not include collecting any participants’ specimens or retrospective information and therefore no participant identifiers were recorded on PT items and tools used. Part of the activities in this study involved health facilities testing the distributed PT materials and therefore signing a confidentiality waiver was done as part of the informed consent. Unfilled confidentiality waiver forms were sent to enrolled facilities before participation and panels were sent to only facilities that signed and returned these forms. All PT testing laboratories incurred no charges for participation and no incentives were given to the testing facilities. The approval to perform the activities in this study was obtained from the Uganda national health laboratory and diagnostics services (NHLDS) review board and permission to publish study findings were sought from NTRL management with approval number SRL/OR-2019003.

## Results

### DTS validation results

There was 100% concordance of MTB and rifampicin susceptibility detection between validation runs done on each of the five selected samples with their expected results from the pretest. The mean cycle threshold (Ct) values for all the samples selected were within the medium-range quantification on Ultra assay as seen in Table 2 below.

**Table 2:** Summary of the validation results (n = 20 per sample).

DTS Sample	*MTB* detection	rifampicin resistance	rpoB1 mean cycle threshold (Ct)	rpoB1 Standard deviation (SD) cycle threshold (Ct)
A-1	Medium	Detected	19.7	1.11
A-2	Medium	Detected	20.2	0.80
A-3	Not detected	Not applicable	0	0
A-4	Medium	Not detected	21.2	0.91
A-5	Medium	Not detected	21.03	1.13

The results above show approximately three (3) cycle threshold cuts when a 1:3 dilution factor was applied to the five selected samples which were pretested using a 1:10 dilution as seen in Table 1. The validation standard deviation for all five DTS sample sets was less than three as shown in Table 2 above.

### Performance of DTS when tested within six weeks

The response rate from participants was 91.5% (215/235) within six weeks. Result reporting completion (for *MTB* and rifampicin resistance detection plus Ct values) was at 85.6% (184/215) with the remaining 14.6% of the participants highlighting difficulty to generate Ct values from the GeneXpert machine The performance of participants has been summarized in Table 3 as shown below.

**Table 3:** Participants’ DTS testing results within six weeks.

Parameter	Indicators	A-1	A-2	A-3	A-4	A-5
TB Detection		n = 215				
	Sites detecting TB n (%)	215 (100%)	214 (99.5%)	2 (1.0%)	214 (99.5%)	212 (98.6%)
	Sites not detecting TB (%)	0 (0%)	0 (0%)	207 (96.3%)	1 (0.5%)	0 (0%)
	Sites reporting uninterpretable TB result*(%)	0 (0%)	1 (0.5%)	6 (2.7%)	0 (0%)	3 (1.4%)
RIF Detection						
	Sites detecting Rif resistance (%)	210 (97.7%)	210 (97.6%)	0 (0%)	0 (0%)	0 (0%)
	Sites not detecting Rif resistance (%)	0 (0%)	1 (0.5%)	207 (100%)	204 (95.4%)	202 (95.5%)
	Sites reporting indeterminate Rif result (%)	1 (0.5%)	1 (0.5%)	0 (0%)	1 (0.5%)	0 (0%)
	Sites not reporting rifampicin detection	4	2	0 (0%)	9	10
Probe values	Probe "rpoB1" Ct mean (SD)	20.1 (3)	22.1 (1.70	3.6 (0.5)	23.9(2.2)	23.2(2.5)

*Uninterpretable result = invalid, error, or no result.

From the results above, there was 97.7%, 97.6%, 96.3%, 95.4% and 95.5% consensus agreement for samples DTS 2019 A-1, DTS 2019 A-2, DTS 2019 A-3, DTS 2019 A-4 and DTS 2019 A-5 respectively among the participants concordant with the expected validation results. The error rate was at 0.93% (10/1075) with the majority of errors reported among *MTB-*positive rifampicin-resistant samples i.e. DTS 2019 A-1 and DTS 2019 A-2. General hospitals recorded the highest number of errors 60%(6/10) while the remaining errors were equally distributed between health center IV and III levels. Seven (7) discordant results were reported from participants with the majority 28.6% (2/7) being for false *MTB* detection and indeterminate rifampicin results individually.

### Performance of DTS panels at varying storage conditions

All sample testing parameters are summarized in Table 4 below.

**Table 4:** Performance of DTS panels tested at different periods and storage temperatures.

Samples tested	Storage temperature And testing time	MTB detection concordance	Rifampicin detection concordance	Median (IQR)	Mean (SD)	P-value	Change in mean Ct value (validation-follow up testing) (°C)
All samples without stratification	Validation (Day zero)	100 (100%)	100 (100%)	20.4 (19.8–21.4)	20.5 (1.2)		
	Within 6wks	1072 (99.7%)	1071 (99.6%)	22.6 (20.7–24.9)	22.8 (3.2)	<0.0001	0.3
	2–8@6 months	80 (100%)	80 (100%)	20.9 (20.6–21.9)	21.1 (1.7)	0.0111	0.6
	RT@6month	80 (100%)	80 (100%)	21.6 (20.8–23.2)	22.2 (2.4)	<0.0001	1.7
	2–8@1 yr	80 (100%)	80 (100%)	21.7 (20.9–23.2)	21.9 (1.9)	<0.0001	1.4
	RT@1yr	80 (100%)	80 (100%)	23.7 (22.7–26.9)	24.9 (3.4)	<0.0001	4.4
	RT@1yr, 2wks in a hot air	80 (100%)	80 (100%)	26.5 (25.1–29.3)	26.9 (2.8)	<0.0001	6.4
A1 (MTB, RIF resistant)	Validation (Day zero)	20 (100%)	20 (100%)	19.7 (18.7–20.5)	19.7 (1.1)		
	Within 6wks	215 (100%)	210 (97.7%)	18.8 (18.1–20.4)	20.1 (3.0)	0.5851	0.4
	2–8@6 months	20 (100%)	20 (100%)	20.9 (20.0–22.1)	21.1 (1.4)	0.0007	1.4
	RT@6month	20 (100%)	20 (100%)	21.8 (20.4–23.0)	21.8 (1.5)	0.0001	2.1
	2–8@1 yr	20 (100%)	20 (100%)	22.4 (21.1–23.2)	22.1 (1.4)	0.0001	2.4
	RT@1yr	20 (100%)	20 (100%)	23.2 (21.4–25.4)	23.5 (2.9)	<0.0001	3.8
	RT@1yr, 2wks in a hot air	20 (100%)	20 (100%)	27.0 (24.8–29.4)	27.1 (2.8)	<0.0001	7.4
A2 (MTB, RIF resistant)	Validation (Day zero)	20 (100%)	20 (100%)	20.0 (19.6–20.9)	20.2 (0.8)		
	Within 6wks	214 (99.5%)	210 (97.6%)	23.1 (21.2–24.3)	22.1 (1.7)	<0.0001	1.9
	2–8@6 months	20 (100%)	20 (100%)	21.3 (20.7–21.7)	20.9 (1.9)	0.161	0.8
	RT@6month	20 (100%)	20 (100%)	21.5 (21.1–21.9)	21.6 (1.2)	0.0018	1.4
	2–8@1 yr	20 (100%)	20 (100%)	21.6 (20.8–22.5)	21.7 (1.9)	0.0066	1.5
	RT@1yr	20 (100%)	20 (100%)	23.5 (21.9–25.6)	24.3 (2.9)	<0.0001	4.1
	RT@1yr, 2wks in a hot air	20 (100%)	20 (100%)	26.4 (25.1–28.7)	26.6 (2.8)	<0.0001	6.4
A4 (MTB, RIF sensitive)	Validation (Day zero)	20 (100%)	20 (100%)	21.3 (20.7–21.7)	21.2 (0.9)		
	Within 6wks	214 (99.5%)	204 (95.4%)	24.1 (22.2–25.2)	23.9 (2.2)	0.0001	2.7
	2–8@6 months	20 (100%)	20 (100%)	21.3 (20.8–22.9)	21.3 (1.9)	0.8053	0.1
	RT@6month	20 (100%)	20 (100%)	21.6 (20.8–23.6)	22.7 (3.2)	0.0718	1.5
	2–8@1 yr	20 (100%)	20 (100%)	21.5 (20.8–22.8)	21.6 (1.9)	0.3505	0.4
	RT@1yr	20 (100%)	20 (100%)	25.8 (23.2–30.1)	26.4 (3.4)	<0.0001	5.2
	RT@1yr, 2wks in a hot air	20 (100%)	20 (100%)	27.7 (25.7–29.4)	27.6 (2.5)	<0.0001	6.4
A5 (MTB, RIF sensitive)	Validation (Day zero)	20 (100%)	20 (100%)	20.9 (20.1–21.9)	21.0 (1.1)		
	Within 6wks	212 (98.6%)	202 (95.5%)	22.9 (22.1–24.4)	23.2 (2.5)	0.0003	2.2
	2–8@6 months	20 (100%)	20 (100%)	20.9 (20.6–21.3)	21.2 (1.5)	0.7979	0.2
	RT@6month	20 (100%)	20 (100%)	21.9 (20.9–23.5)	22.8 (2.9)	0.0312	1.8
	2–8@1 yr	20 (100%)	20 (100%)	21.6 (21.0–23.4)	22.4 (2.1)	0.0248	1.4
	RT@1yr	20 (100%)	20 (100%)	24.6 (23.4–27.7)	25.8 (3.8)	0.0001	4.8
	RT@1yr, 2wks in a hot air	20 (100%)	20 (100%)	25.9 (24.0–28.8)	26.5 (3.2)	<0.0001	5.5

Validation; testing done immediately after PT preparation, Within 6wks; testing done within six weeks after panel preparation, 2–8@6months; testing samples stored at 2–8°C at six months, RT @6months; testing samples stored at room temperature at six months, 2–8@1 yr; testing samples stored at 2–8°C after one year, RT@ 1yr; testing samples stored at room temperature for a year, RT @1 yr, 2wks in hot air; testing samples stored at room temperature for a year and then incubated at 55 in a hot air oven.

From Table 4 above, DTS PT precision (determined by standard deviation) within six weeks of testing by participants varied slightly above the set target of 3.0 from the validation results. Without sample stratification, the overall highest mean Ct value increase was seen after one year of storage at room temperature by a difference of 4.4. Heating the DTS samples at 55°C for two weeks after a year of storage further increased the overall mean Ct by a difference of 6.4. Testing done at six months on storage at 2–8°C showed no statistical difference with the validation results. At all the remaining testing times and conditions, P-values were less than 0.008 although the absolute mean Ct when compared showed slight increments and accommodated differences for the detection of *MTB and* rifampicin susceptibility.

There were no false negative, false positive, or false rifampicin susceptibility detection or uninterpretable results reported after six months at the different storage temperatures. There was no statistically significant difference in deterioration between the sensitive and the resistant samples used in this study. The DTS panels produced according to this methodology were homogenous and accurate, achieving 100% test result concordance with parental pre-test stock results for the detection of *M*. *tuberculosis* and rifampicin resistance giving 100% specificity and sensitivity of the DTS method. With sample stratification, Sample DTS 2019 A-4 had the highest mean Ct value increase of 5.2 compared to other samples. From Table 2, DTS 2019 A-4 had the highest mean Ct value and this could contribute to the higher increase after one year of storage compared to other samples which had slightly lower mean Ct values.

The overall concordance for the detection of *M*. *tuberculosis* and rifampicin resistance between DTS panels tested by the participants within six weeks and the parental stocks was 95.6%. This showed that the DTS preparation method used was appropriate and testing instructions were user-friendly. The seven (7) discordant results recorded at week six were from five (5) sites of which three were general hospitals and the reminder national and regional referral hospitals. Of the discordant results registered, one false positive and negative were due to the switching of samples during testing at the facilities. Two sites that reported indeterminate results stated having experienced accidental pouring of the diluted DTS samples and further re-diluting the remaining portion to make runs on the Ultra assay. The two indeterminate results obtained for samples DTS 2019 A-1 and DTS 2019 A-4 showed outlier rpoB1 Ct values of 31 and 34 and SPC Ct values of above 34 respectively. This finding agrees with other quantitative research assessments that have associated high Ct values with false rifampicin detection [12,17].

**Fig 1:**
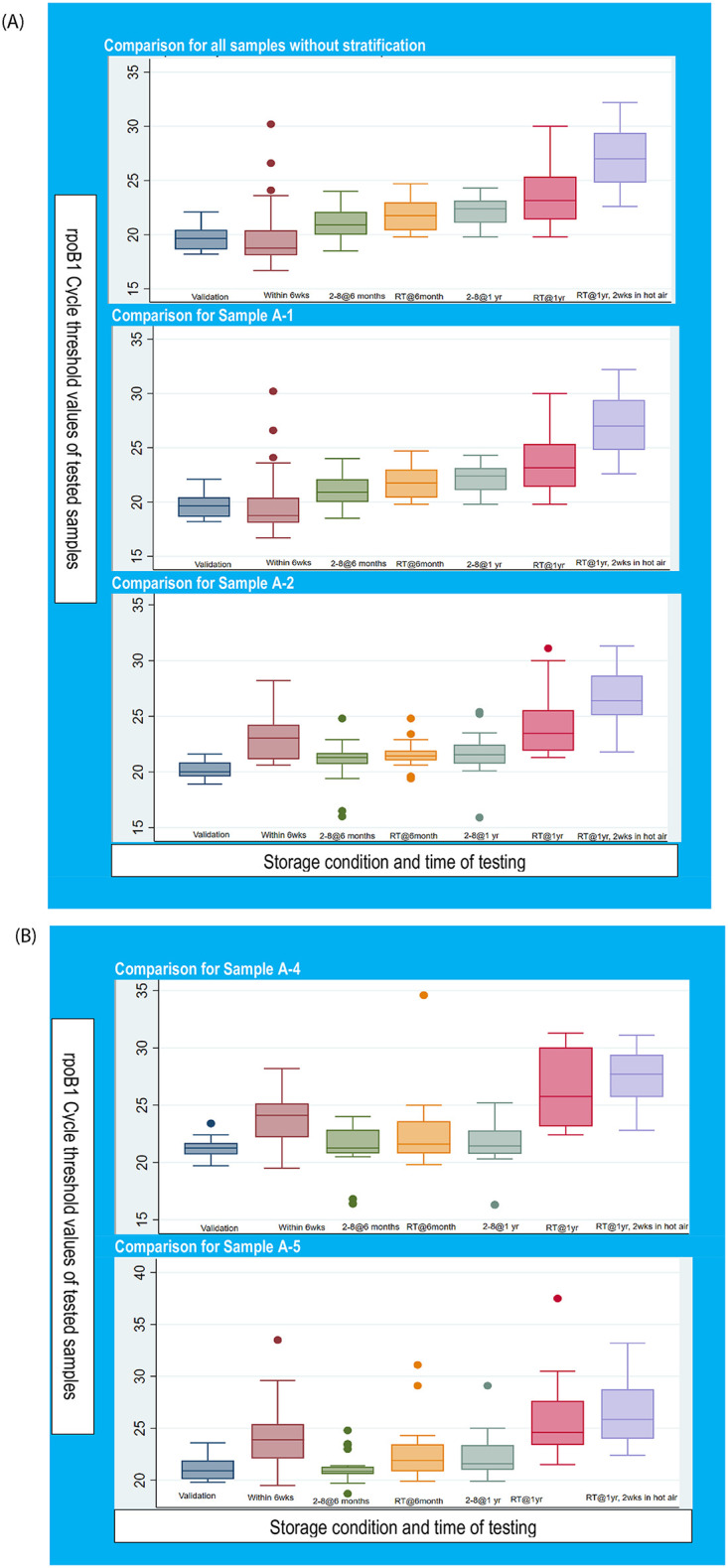
Boxplot of rpoB1 Ct values of DTS tested at the different temperatures and periods.

For result visualization, the outcome of this study was plotted on boxplots as shown in Fig 1. The graph highlights results with and without stratification for the DTS samples to ascertain changes in sample stability.

The resulting assessment without sample delamination showed a narrow difference in the median value of cycle threshold for samples tested at validation, at six months for both storage conditions and at 2–8°C stored for a year. The box plotting for samples stored at room temperature and then two weeks incubation at 55°C showed a higher median than the rest of the other test conditions. Outliers were observed with testing at validation, room temperature at six months and after one year at both conditions nevertheless these affected less the median values and individual value distribution around the median.

The boxplot for all DTS samples prepared showed medians within close ranges at all testing conditions apart from testing after one year of storage at room temperature and two weeks of heating at 55°C.

All the plots showed narrow box plot ranges and shorter whisker lengths throughout both which increased with increasing storage time. The boxplots become broader for DTS stored at room temperature showing higher median values compared to DTS at 2–8°C as seen in Table 4 and Fig 1 above.

## Discussion

We found that DTS samples stored at 2–8°C remain reliably more stable and can effectively be used as PT samples for up to one year of storage. Our study did not observe any trends between the strain used to produce DTS and the proportion, types of discordance and errors obtained at testing sites. From our findings, we noted that DTS with a higher mean Ct value at validation will deteriorate faster compared to samples with slightly lower initial stock Ct value reading. Of the entire ten (10) error results reported, 50% (6/10) were “no results” that were caused by a power blackout during DTS running on the Xpert machine. The other errors reported in this study were 5006 associated with improper sample volumes and reconstitution. Error codes 2027, and 1001 associated with communication loss between the Xpert machine and the Ultra software on the computer and high surrounding temperature respectively were also reported in this study [18].

The standard deviation variation among samples tested in this study was less than three (<3) which we considered as being a high level of homogeneity and consistency of DNA concentration in the samples. These findings highlight consistency in DTS integrity as also highlighted by Kyle *et al* [12]. The variations in SD values for the different samples can be due to differences in reconstitution volumes used by participants who ran the test with varying materials and equipment at their respective sites.

Overall Comparison between samples stored at room temperature and 2–8 highlights a higher variation among room temperature DTS since under routine room temperature storage, consistency of temperature is hard to achieve. The mean Ct value increase of 4.4 among unstratified samples further showed that room temperature conditions affect and decrease the concentration of *MTB* DNA faster compared to when the samples are stored at 2–8°C. Further exposure of the samples at 55°C for two weeks highly degraded the samples and had more scattered Ct values. This is consistent with molecular biology concepts that highlight the degradation of DNA with increasing temperature exposure lowering DNA concentration and decreasing DTS stability [19]. There was no statistical difference in the stability of both the rifampicin-sensitive and resistant samples measured as a change in Ct values. This shows that both rifampicin-sensitive and resistant *MTB* strains are affected equally by temperature and storage time.

## Conclusion

Dried Tube Specimens stored at 2–8°C remain reliably more stable for one year compared to higher temperatures and can be consistently used as PT materials in more than one PT round where a provider can prepare a bulk of DTS at once. This finding suggests a change in DTS preparation processes for PT providers who enroll biannual schedules creating room to prepare PT panels once per year. Scheming the preparation procedure this way with storage conditions of DTS maximized at 2–8°C helps to minimize the resources required for Ultra PT provisions such as equipment, reagents, and time. This when applied in resource-limited countries promotes the implementation of highly reliable, consistent, sustainable, and continuous PT programs.

## Supporting information

S1 File Standard operating procedure for GeneXpert PT preparation. (PDF)

S2 File Raw data excel sheet. (XLSX)
