# Supplementary material for: Advancing proficiency testing for ultra in resource-limited settings using dried tube specimen: A study by SRL-Uganda
Source: PLoS One. 2023 Mar 10;18(3):e0282650. doi: 10.1371/journal.pone.0282650 (PMC10004543; doi:10.1371/journal.pone.0282650)
Supplement: S1 File — (PDF) [file pone.0282650.s001.pdf]

One PT panel retained at NTRL is tested at least two weeks by doing LPA analysis.

The LPA results must score 100% of expected results on both INH and RIF drugs.

6.4.7 Storage and transport

See SOP PT 012 Handling and Storage of PT items.

6.4.8 Retaining of PT items

- Prepare and retain at least two (02) complete PT sets for each round
- Follow steps below Section 6.7

6.5 Genotypic DST: Preparation of Dried Tube Specimen Xpert MTB/RIF

6.5.1 Materials needed

Standard control strains of *Mycobacterium tuberculosis*, well characterised strains from the Uganda NTRL/SRL repository (PT 009 F28 DTS for Xpert MTB/RIF Repository form). **Select 8 per start point.**

- Phosphate buffered saline (PBS)
- Sterile water (distilled and autoclaved)
- Xpert MTB/RIF / Ultra assay kit (Cepheid)
- Blue food colouring
- BACTEC MGIT 7ml tubes (BD)
- BACTEC MGIT PANTA supplement(BD)
- LJ plain slants
- 4ml and 2ml cryovials
- Cryo-vials labels
- Disinfectant (5% Lysol, 1% bleach and 70% alcohol)
- 3 mm glass tubes, 50ml conical tubes, sterile 16 x 100 mm glass tubes with screw caps, sterile disposable loops and pipette tips; 20µl, 200µl and 1000µl
- GeneXpert Dx System (GeneXpert machine, computer, barcode scanner and printer)
- Biosafety cabinet class II
- N95 Respirators
- Timers
- Trolley cart
- Vortex mixer
- Micro centrifuge tube racks
- Eppendorf's repeater plus pipette
- Refrigerated centrifuge
- Racks (for 50 ml conical tubes and 16mm tubes)
- Automatic pipette (P 1000, P200, P20 and P20)
- BD MGIT 960 instrument
- Middlebrook 7H9 broth
- Plastic bio-transport container

- Plastic rack for 2-ml cryovials
- Plastic cryoboxes for 2-ml cryovials
- Pipette aids, Bench liners, Lysol: Prepare working solution daily (final concentration of the active ingredient, sodium hypochlorite or chlorine, is no less than 0.5%), 70% ethanol, Biohazard discard bags, Disposable 10 µl loop-sterile, serological pipettes (10ml and 25ml), Disposable nitrile gloves, 3-ml graduated sterile transfer pipettes.

Source

Standard control strains of *Mycobacterium tuberculosis*, well characterised strains from the Uganda NTRL/SRL database repository PT 009 F28 DTS for Xpert MTB/RIF Repository form

1. MTB strains with know resistant pattern for RIF and INH (through molecular typing either done in Antwerp SRL or at NTRL Uganda) will be used for preparation of all panels.(RIF resistant *M. tuberculosis* with different mutations in the *rpoB* gene associated with RIF resistance. **Do not select MDR MTB** (i.e., resistant to INH and Rifampin).
2. Susceptible strains to both RIF and INH to be used will be standard strain of H37R*v* or other well chorarcterised available at the Uganda NTRL
    a. *NB: Refer to 6.3.2 for more details*
3. Non Tuberculosis mycobacterium (NTMs) for the Negative panels.

Record selected strains on (PT 009 F24 DTS Preparation for Xpert MTB/RIF Worksheet)

NB: Preparation of strains for should begin at least 3 months to allow for adequate time for MGIT culture

i. culture of selected strains on MGIT for maximum of 3 weeks inactivated then viability checked wiith MGIT culture.

6.5.2 Preparation procedure

Note: *Determine procedure workflow and schedule to complete all procedure steps. Prepare and label materials (i.e., media, reagents, supplies) needed to move from one procedure step to another. Preparation and labeling of materials may be done in a BSL2 space. Move labeled materials to the TB containment room (using a laboratory cart) where manipulation of cultures and isolates are done. Record media and reagents lot number and expiration dates on the various forms. See attached forms*

6.5.2.1 Procedure for culture isolates

Grow reference strains in broth culture (MGIT tube)

1. Select at least eight strains of mycobacterial species from the permanent stocks in freezer vials. Check the repository PT 009 F28 DTS for Xpert MTB/RIF Repository form
    - strains of pan susceptible MTB (atleast 2 strains)
    - strains of RIF resistant MTB (atleast 2 strains)
    - species of NTM (atleast 2 strains)
    - By the end of the DTS preparation procedure, five strains will be selected for preparation of PT panels. Each panel will contain five DTS sample tubes

2. Record isolate name and complete all needed information on the DTS Stock Preparation Form **PT 009 F24 DTS Preparation for Xpert MTB/RIF Worksheet.**
3. Label 4 MGIT tubes for each MTB isolate. Label 4 MGIT tubes for Artificial sputum (total of **32** MGIT tubes).

1. Perform all mycobacerial culture manipulation in the BSL 3 biosafety cabinets class 11
2. Clean the work surface and walls of the the BSC using tissue paper soaked with 5% lysol.
3. Set up the workspace in the BSC by placing an absorbent line on the work surface and soak it with 5% lysol.
4. Place rack of sterile MGIT tubes to be innoculated, “seed cultures” from the repository to transfer or vial from freezer storage, rehydrated PANTA supplement, 1 ml graduated, sterile transfer pipettes on the surface of the BSC.
5. Add 0.8 ml of PANTA supplement to each sterile MGIT tube using an automatic pipette reserved for reagents only.
6. Innoculate 0.25 ml of DTS isolate seed culture into MGIT tube with PANTA supplememt using automatic pipette.
   Note*:* Work with one tube at a time to avoid cross-contamination.

7. Replace all caps tightly and gently invert freshly innoculated MGIT tubes 2-3 times to mix.
8. Record all samples and neccesary information on form A005 F1 MGIT inoculation worksheets
9. Enter all innoculated tubes into the MGIT 960 machine
10. When the MGIT tube flag positive on MGIT machine 960 , unload and place them on the rack.
11. Remove positive tubes and check for appearance consistence with *mycobacterium tuberculosis* complex (MTBC) or other *Non-Tuberculosis mycobacteria* (NTM).
12. Record the date of positivity on each tube, the expected date of inoculation out date and intials
13. Print the positive report and write the date of MGIT positivity on the MGIT tube.
14. Incubate all cultures 5 days past MGIT positive date at 37° C before storing at 2-8 °C.
15. After the 5 days incubation, store MGIT cultures at 2-8ºC while waiting for the other cultures to flag positive. The wait should not be longer than 30 days.

6.5.2.2 Check for purity of mycobacterial culture

1. Place rack of positive MGIT cultures inside the BSC.
2. Set timer: Vortex each tube for one minute. Allow tubes to stand for 10 minutes.
3. See the form A005 A1 for MTB identification alogarithm
4. Inoculate each sample on Blood agar plate to check for contaminats. Incubate at 37°C for 24 hours.
5. Remove the plate(s) from the incubator, read the BA results and record the results/date on Form PT009 F24 Preparation of DTS worksheet.

6. Once the there is no growth on the blood agar, perform SD TB MPT 64 Rapid test (for confirmation of *M.tb* complex strain).
7. Record results/date on Form PT009 F24 Preparation of DTS worksheet

Note: **If positive results are obtained report, MTB complex**

8. Label 2 clear LJ slants with the corresponding isolates. Perform all mycobacterial culture manipulation in the BSL 3 laboratory.
9. Vortex each culture several times to mix. Allow to settle undisturbed for 20 minutes.
10. Prepare a 1:100 dilution of each isolate with sterile distilled water.
11. Innoculate 100µl (2-3 drops of the isolate) of the 1:100 dillution onto 2 LJ slopes.
12. Discard pipette tips into pipette discard with 5% lysol.
13. Incubate plates at (35-37)°C for 1-3 weeks Note: Rapidly growing NTM will exhibit good growth in one week and MTB will grow in 2-3 weeks.
14. Record the innoculation date on form "PT 009 F24 DTS Preparation for Xpert MTB/RIF Worksheet"
15. After 3 weeks qautify the colonies on each slant.
16. Record results on form "PT 009 F24 DTS Preparation for Xpert MTB/RIF Worksheet

Note: *Interpretation of purity check plates*: Incubate plates for up to 3 weeks. Check plates for growth at least once per week. Examine growth to confirm that the morphology of the colonies is consistent with the selected species of mycobacteria. Discard MGIT (or stock) cultures that grow mixed cultures on the LJ slants and/or colonial morphology is not consistent with the expected morphology for the selected species of mycobacteria. Record results on the Form "PT 009 F24 DTS Preparation for Xpert MTB/RIF Worksheet"

Note: Work for Steps **6.5.2.3 – 6.5.2.5** may be performed in one day. If these steps cannot be completed in one day, work may be spread over 2 days; however, Steps 11.4-11.6 **must** be completed on the same day. Example of work schedule:
Day 1 – perform step 6.5.2.3 (heat-inactivate culture) then store inactivated MGIT cultures at 2-8°C.
Day 2 – perform Steps 6.5.2.4-6.5.2.5

6.5.2.3 Inactivation of Mycobacterial cultures

6.5.2.3.1 Heat -inactivate culture to kill the mycobacteria

1. Heat-inactivate all positive MGIT cultures at the same time.
2. **2.** Check oven temperature reading is at 80-85°C.
3. **3.** Place rack of MGIT cultures inside the oven.
4. **4.** Close oven door tightly and wait for temperature to stabilize at 80-85°C.
   Note: Oven temperature typically drops when door is opened; wait for temperature to reach 80°C before starting the timer.
5. **5.** Start timer for 30 minutes. Record oven temperature and time on the inactivation verification worksheet.

6. After 30 minutes has elapsed, verify oven temperature is 80-85°C. Record temperature and time on the PT 009 F25 DTS Inactivation Verification Form. Do not open the oven.
7. Set timer for additional 30 minutes.
8. Once a total of one hour has elapsed, verify temperature is 80-85°C. Record temperature and time on the PT 009 F25 DTS Inactivation Verification Form.
9. Remove rack of MGIT culture tubes from the oven.
10. Allow tubes to cool to room temperature. Label rack "heat-inactivated cultures" and date of inactivation.
11. Proceed to the next step (6.6.2.4) to prepare stock solutions of the heat-inactivated cultures.

6.6.2.3.2 Prepare stock solution of the heat- inactivated culture

1. Place rack of inactivated MGIT culture tubes inside the BSC. Make sure the cultures have been allowed to cool to room temperature.
2. For each MGIT culture, prepare one tube containing 5-10 sterile 3 mm glass beads.
3. Add the glass beads to the MGIT culture tube. Work with one culture tube at a time to avoid cross-contamination.
4. Gently remove the cap from the inactivated MGIT culture tube.
5. Hold MGIT tube at 45-degree angle and slowly pour in 5-10 pieces of sterile 3 mm glass beads while holding the mouths of the two tubes together. Discard the bead tube in designated waste container (do not re-use).
6. Tighten cap of the MGIT tube and vortex tube for 5 minutes. Make sure a full vortex is obtained.
7. Allow tubes to sit for 10 minutes undisturbed, for clumps to settle.
8. Label one 50-ml sterile conical tube for each isolate.
9. Using sterile transfer pipette, carefully pipet off all the liquid above the beads and transfer fluid to the labeled 50-ml conical tube. Work with one culture tube at a time to avoid cross-contamination.
10. Combine the liquids from the same isolate in the same conical tube.
11. The combined suspensions of each isolate will be the **stock solution** to use for performing inactivation verification and for preparing dilutions.

    Note: At this point, there will be eight (8) conical tubes of stock solutions, one for each of the eight isolates selected at the beginning of the procedure.
12. Label each tube of the stock solutions with the assigned stock number (e.g. DTS 2019 01 corresponding to stock #1 for year 2019). **F24 DTS Preparation for Xpert MTB/RIF Worksheet.**
13. Proceed **immediately** to the next step (6.6.2.4) to check viability of the stock solutions of each isolate.

6.5.2.4 Viability Check of mycobacteria in the stock solution for inactivation verification

1. Prepare one MGIT tube for each heat-inactivated stock solution. Label tubes with stock solution number, name of the isolate, and date of inoculation.
2. Using a repeat pipettor, add 0.8 ml of MGIT supplement to each MGIT tube. Dont add the PANTA componet.
3. Invert heat-inactivated stock solution 2-3x to mix.
4. Using 1-ml sterile transfer pipette, transfer 0.5 ml of the stock solution of each isolate to its labeled MGIT tube. Cap tightly and invert MGIT tube 2-3 times to mix. Work with one stock solution at a time to avoid cross-contamination.
5. Load inoculated MGIT tubes in the MGIT instrument.
6. Leave tubes in the instrument for two 42-day cycles (total of 84 days) or until flagged positive by the MGIT instrument.
7. Scan out negative tubes when the first 42-day cycle is completed. Print Unloaded Negative Report from the MGIT instrument. File report in the DTS Preparation binder.
8. Scan tubes back into MGIT 960 instrument to start the second 42-day cycle.
9. Scan out negative tubes when the second 42-day cycle is completed. Print Unloaded Negative Report from the MGIT instrument. File report in the DTS Preparation binder.
10. Scan out positive MGIT tubes, if any. Print Unloaded Positive report. File report in the DTS Preparation binder.
11. Discard the stock solution for any viability test that is flagged positive by the MGIT instrument. Growth in MGIT tube indicates that the heat-inactivation procedure failed and the organism is still viable and must not be used to prepare final DTS panels.
12. Record MGIT results for each 42-day cycle of the viability check on form PT 009 F25 DTS Inactivation Verification Form
13. Proceed **immediately** to the next step (6.6.2.5) to prepare dilutions of the stock solutions for DTS pre-testing.

6.6.2.5 Prepare dilution of the stock solution of the isolates for DTS pre-test

1. Prepare materials and place inside the BSC.
2. Five cryovials (4-ml capacity) for each stock solution. Label each vial with name of isolate, stock number, and aliquot number or letter (A-E).i.e. H37Rv DTS 9001 01 A
3. Eight 50-ml sterile conical plastic centrifuge tubes, label with name of isolate, stock solution number, dilution (1:10), and date.
4. Prepare a 1:10 dilution of each heat-inactivated stock solution.
5. Pipet 4.5 ml of sterile saline to each labeled 50-ml conical tube.

6. Add 5 µl of food coloring to each 50-ml conical tube.
7. Using an automatic pipet , transfer 0.5 ml of stock solution to the labeled 50-ml conical tube. Vortex tube for 30 seconds. Work with one stock solution at a time to avoid cross-contamination. Allow tubes to stand for 10 minutes after vortexing (set timer) to allow for settling of large clumps to achieve a more homogenous aliquots.
8. Prepare 5 DTS tubes (4-ml cryovials for each of the 1:10 diluted stock solution.
9. Uncap labeled 4-ml cryovials inside the BSC and place caps in a clean plastic zipper bag.
10. Using a repeat pipettor, pipet 100 µl of the diluted stock into the 5 labeled cryovials. Keep pipette tip in upper 1/3 of the fluid when aspirating the diluted stock to avoid disrupting larger clumps that settled to the bottom of the tube. Work with one diluted stock at a time to avoid cross-contamination.
11. Discard the remaining 1:10 dilution.
12. Store remaining stock solutions in the refrigerator set at 2-8°C.
13. Allow aliquot tubes to sit open inside the BSC class 11 in the BSL3 containment laboratory for 7-10 days.
14. After 7-10 days, check if specimen at the bottom of the tubes is dry, then tightly cap all tubes. Ensure that tubes are visually dry before capping.
15. Store DTS in the dark at room temperature while waiting for pre-testing.
16. Proceed to next step (6.6.2.6) for DTS pre-testing.

6.6.2.6 Perform DTS pre-test to determine which stock solutions to include in the DTS panel

1. Test the five aliquots from the 1:10 dilution of each stock with Xpert MTB/RIF / Ultra assay following this procedure see SOP A010 for Genexpert assay
2. Add 2.5 ml of SR to each DTS sample to be tested. Tightly recap sample
   Note: Work with one DTS at a time to avoid cross-contamination and process only samples to be tested in that round. Shake vigorously 20 times. One back and forth movement is a single shake.
3. Incubate the samples for 10 minutes (set timer) at room temperature.
4. Shake the samples vigorously again 20 times.
5. Incubate for an additional 5 minutes (set timer) at room temperature.
6. Add 2.0 ml of the sample to labeled Xpert MTB/RIF cartridge. Scan and load cartridges in the GeneXpert instrument.
7. Once pre-testing is complete, print test reports.
8. From the printed test results, enter pre-test data into the **DTS Pre-test PT 009 F24 DTS Preparation for Xpert MTB/RIF Worksheet.**
9. Proceed to next step (6.6.2.7) to prepare the PT panel.

Note: Pretest can be done for any of the Genexpert assay (i.e. MTB/RIF or Ultra assay) since the objective is to predict the concentration that will be used to prepare the final panel

6.6.2.7 Aliquot DTS samples to prepare DTS final panel

1. Using data from the DTS **PT 009 F24 DTS Preparation for Xpert MTB/RIF Worksheet**, determine which heat-inactivated stock solutions to include in the DTS panel. Select stocks based on these criteria: Using data from the DTS PT 009 F24 DTS Preparation for Xpert MTB/RIF Worksheet, determine which heat-inactivated stock solutions to include in the DTS panel. Select stocks based on these criteria:
   - With a mean cycle threshold (Ct) for Probe A and IS 6110 in the medium to low range (16-23 Ct) and a standard deviation ≤ 3.
   - With 100% accuracy of results (i.e., all 5 aliquots for each isolate gave the expected result).

Isolate	Expected result
Pan susceptible MTB	MTB detected; RIF resistance not detected
RIF resistant MTB	MTB detected; RIF resistance detected
Negative	MTB not detected

2. Select five inactivated stock solutions.
3. Do not use stocks that failed the purity check and heat inactivation verification or those that did not meet the above criteria
4. Record stock number of the solutions selected for DTS panel on PT 009 F34 DTS Stock selection log.
5. Determine the number of 4-ml cryovials (aliquots) needed per stock. Add at least 15% extra for panel validation and for other needs like for QC and instrument verification.

   For example: If there are 78 sites enrolled in the PT program, then 78 panels are needed.

   78 x 15% = 11.7 (round up to 12) 78 +12 = 90 (at least 90 aliquots must be prepared to ensure enough aliquots are available for validation and for all the 78 testing sites enrolled in the Xpert MTB/RIF PT program).

   There are 5 stock solutions to aliquot: 90 tubes per stock x 5 = 450 total number of cryovials needed.

   Place tubes in racks and label racks with isolate name and stock number.
6. Prepare all materials and place them inside the BSC in the BSL3 containment laboratory.
7. Select type of dilution to use for DTS sample aliquots. There are two options to dilute stocks for DTS aliquots.
   - **Use 1:10 dilution of inactivated stock solution when mean Ct for Probe A and IS 6110 for Ultra is 16-17 on pretest**. Refer to Table 1 for calculations

Table 1. Preparation of 1:10 Dilutions (example of calculations for 250 and 400 aliquots)

**A.** Number of aliquots per stock	**B.** Total volume of 1:10 diluted stock required **[(A x 0.1* ml) + 5 ml]**	**C.** Volume of stock solution vortexed with beads **[(B/10) + 0.5 ml]**	**D.** Volume of vortexed stock added to saline for 1:10 dilution **(B/10)**	**E.** Volume of saline for 1:10 dilution **(B-D)**	**F.** Volume of food grade dye for 1:1000 dilution **(B/1000)**
250	25 ml + 5 ml = 30 ml	3.0 + 0.5 = 3.5 ml	3 ml	27 ml	30 μl
400	40 ml + 5 ml = 45 ml	4.5 + 0.5 = 5.0 ml	4.5 ml	40.5 ml	45 μl

***0.1 ml (10**

*01 ml (100 μl) is the volume of diluted stock solution aliquot for each DTS tube

- **Use entire volume of inactivated stock solution when mean Ct for Probe A or lS 6110 for Ultra is 18-23 on pre-test**. Dilute stock with saline only enough to produce the desired number of aliquots plus 5 ml for pipetting error. Refer to Table 2 calculations.

Table 2. Dilution of entire stock calculations (example of calculations for 250 and 500 aliquots)

A. Number of aliquots per stock	B. Total volume of diluted stock required [(A x 0.1* ml) + 5 ml]	C. Volume of stock solution	D. Volume of stock added to saline	E. Volume of saline for dilution (B-D)	F. Volume of food grade dye for 1:1000 dilution (B/1000)
250	25 ml + 5 ml = 30 ml	Entire volume of stock __ ml	Entire volume of stock above beads (9 ml)	30 ml – 9 ml = 21 ml	30 ml/1000 = 30 μl
500	50 ml + 5 ml = 45 ml	Entire volume of stock __ ml	Entire volume of stock above beads (9 ml)	55 ml – 9 ml = 46 ml	55 ml/1000 = 55 μl

*0.1 ml (100 μl) is the volume of diluted stock solution aliquot for each DTS tube

8. Prepare dilution of stock solutions. Note: The type of dilution must first be selected
9. Transfer the required volume of stock solution (C) to a labeled sterile 16 mm x 100 mm tubes with 5-10 sterile 3-mm beads.
10. Vortex each tube for 5 minutes ensuring a full vortex is obtained.
11. Allow tubes to stand for 10 minutes undisturbed.

12. Transfer the required volume of vortexed stock (D) from above the beads to a 50-ml plastic conical tube. Do not push pipette tips into the beads or disturb the beads.
13. Add required volume of sterile saline (E) to the tube. Depending on total volume, dilutions may need to be split between two 50-ml conical tubes.
14. Add blue food coloring at a concentration of 1:1000 to each tube. Refer to Table 1 and Table 2 for the appropriate volume of the food coloring.
15. Vortex each dilution tubes for 30 seconds.
16. Remove caps from cryovials inside the BSC and place caps in plastic zipper bag. Write the stock number and isolate name on the bag.
17. Using a repeat pipettor, aliquot 100 μl of each diluted stock solution to the 4-ml cryovials. When aspirating solution, always keep pipette tip in upper 1/3 of the solution to avoid disrupting larger clumps that have settled to the bottom of the conical tube. Always prime pipette 2-3 times by expressing back into dilution tube before beginning to aliquot.
18. Return any remaining stock to storage at 2-8°C.
19. Allow aliquot tubes to sit open inside the BSC in the BSL3 laboratory for 5-10 days.
20. After 5-10 days, check if specimen at the bottom of the tubes is dry, then tightly cap all tubes. Ensure that tubes are visually dry before capping.
21. Place all DTS aliquot tubes in labeled large plastic zipper bags. Store at room temperature in the dark until validation testing. DTS tubes must remain inside the TB containment laboratory until the inactivation verification is completed and passed.
22. Determine and randomly select the number of aliquots to be used for DTS panel validation. Perform panel validation testing (6.6.2.8).
23. Check viability test results for the stock solutions. If stock solutions have passed the viability test (i.e., no growth is detected after 84 days), and the laboratory supervisor has reviewed and signed the PT 009 F25 DTS Inactivation Verification Form, the DTS aliquots may be brought into the BSL2 area for labeling, packing, and storage.
24. Check panel validation results. If panel validation passed, proceed to labeling and packing of DTS aliquots.

6.6.2.8 DTS Panel Validation

1. When preparing DTS for the first time, validate 10% of the prepared aliquots.
2. Once data has been collected from 3 previous panels, use data to calculate the mean standard deviation. Use Table 3 to determine the number of aliquots (sample size) needed to perform a robust validation according to the historical variability observed in the previous 3 panels.

Table 3. Sample Size Calculation

**One Variance Power Analysis**					
Numeric Results when H0: S0 = S1 versus Ha: S0 ≠ S1					
**Power**	**N**	**SO (Standard deviation)**	**SI**	**Alpha**	**Beta**
0.95223	7	1.0000	3.0000	0.05000	0.04777
0.95094	8	1.1000	3.0000	0.05000	0.04906
0.96256	10	1.2000	3.0000	0.05000	0.03744
0.95403	11	1.3000	3.0000	0.05000	0.04597
0.95519	13	1.4000	3.0000	0.05000	0.04481
0.95133	15	1.5000	3.0000	0.05000	0.04867
0.95232	18	1.6000	3.0000	0.05000	0.04768
0.95459	22	1.7000	3.0000	0.05000	0.04541
0.95562	27	1.8000	3.0000	0.05000	0.04438
0.95386	33	1.9000	3.0000	0.05000	0.04614
0.95203	41	2.0000	3.0000	0.05000	0.04797
		2.1000	3.0000	0.05000	
		2.2000	3.0000	0.05000	
		2.3000	3.0000	0.05000	
		2.4000	3.0000	0.05000	
		2.5000	3.0000	0.05000	
		2.6000	3.0000	0.05000	
		2.7000	3.0000	0.05000	
		2.8000	3.0000	0.05000	
		2.9000	3.0000	0.05000	
		3.0000	3.000	0.05000	

3. Each year thereafter, re-calculate the sample size needed for validation using the mean standard deviation of probe A (for all samples positive for MTBC) from the previous three panels. See table below for example. **Example:** If the mean standard deviations for Probe A for all MTBC positive samples from the previous three panels are equal to 1.09, 1.73, and 1.83 the mean standard deviation for probe A for all 3 panels would equal 1.55. Round 1.55 to 1.6. Find 1.6 in the S0 column of the Table 3.The number of aliquots (N) to test is 18. For this year, 18 samples would be tested for validation, regardless of the number of aliquots or panels prepared.

Table 4. Example of Sample Size Calculation

A. Mean SD of I for 2016-C Pane	B. Mean SD of I for 2017-A Pane	C. Mean SD of for 2017-B Pan	D. Mean SD last 3 panels	E. Correspondin sample size acco

				to table
(2016-C-1 SD +2016-C-2 SD +2016-C-3 SD +2016-C-5 SD*) = Mean SD for [illegible]	(2017-A-1 + 2017-A-2 +2017-A-3 +2017-A-4*) / 4 = Mean SD for 2[illegible]	(2017-B-1 + 2017-B-2 + 2017-B-4 + 2017-B-5*) / = Mean SD for [illegible]	(A + B + C) [illegible] = Mean of th[illegible] mean SD fro[illegible] previous 3 pa[illegible]	1.55 (D) rounded[illegible] one decimal poin[illegible] is 1.6
(1.15 + 1.01 +1.09 +1.12) / 4 = 1.09	(1.35 + 2.54 + 1.87 + 1.17) / 4 = 1.73	(1.79 + 1.64 + 1.90 + 1.99) / 4 = 1.83	(1.09 + 1.73 + 1.83) / 3 = 1.55	Find 1.6 in chart and see correspo[illegible] value in N colum[illegible]
*2016-C-4 samp[illegible] a NTM	*2017-A-5 samp[illegible] a NTM	*2017-B-3 sam[illegible] a NTM		

4. Perform DTS panel validation
5. Randomly select the 10% or number of DTS aliquots calculated from Table 3.
6. Label selected DTS aliquots with the name of the isolate, stock number and serial number. Example: H37Rv-1801-1, H37Rv-1801-2, H37Rv-1801-3… (Up to 18, the calculated sample size (N) from the example above)
7. Test samples. Follow procedure for Xpert MTB/RIF testing of DTS samples.
8. Repeat steps 4 to 6 for Xpert MTB/RIF Ultra
9. If the GeneXpert reports an uninterpretable result (error, invalid, or no result), repeat testing with a new aliquot of DTS sample.
10. Once validation testing of the DTS panel is completed, print test reports.
11. Enter all results for both assays on the Xpert MTB/RIF PT 009 F27 DTS for Xpert MTB/RIF. The mean cycle threshold (Ct), standard deviation (SD), and % CV for each probe will be calculated for each validated sample.

6.6.2.9 Calculation

1. Organism qauntification LJ culture count.
2. After incubation at 37 °C for 3 weeks remove plates and count colonies. Colonies counted x 10^4 = approximately CFU/ ml
3. Enter all results on the PT 009 F24 DTS preparation for Xpert MTB/RIF worksheets.
4. Calculation from the Xpert instruments are performed by the GeneXpert sytem from the measured fluorescent signals and embedded calculation algorithms. Lower Cycle Threshold (Ct) values represent a higher starting concentration of DNA template; higher Ct values represent a lower concentration of DNA template.
5. Panel validation calculations; enter all results onto PT 009 F27 DTS Xpert MTB/RIF panel validation worksheets.
6. For each sample, calculate the cycle threshold (Ct) mean, standard deviation (SD), and %CV for each probe.

6.6.2.10 Interpretation/ Results

1. Test results refer to SOP AO10 GeneXpert MTB/RIF Assay.
2. Panel validation results;
   a. The level of detection result target is low-medium range (16-28) Ct.
   b. The sample is considered valid if the calculation coefficient of variation (% CV) for probes not detecting resistance in the isolate is ≤10, SD ≤3 and sample falls within the expected range of the semi-qauntitive results as listed in the table 2 (low to medium).
   c. In certain rifampin-r/#]
   d. esistance isolates, a probe may exhibit incomplete fall-out i.e, the probe fails to bind (Ct =0) in most but not all instances. The %CV for these probes is not a true reflection of the variability in the sample preparation.

Table 5: Xpert MTB/RIF or Ultra semi-qauntitative results,

MTB results	Ct range
High	Below 16
Medium	16-22
Low	22-28
Very low	Above 28

6.6.2.11 Troubleshooting

1. Increased cycle threeshold results If the SPC internal control cycle threshold result for a single aliquot is ≥ 34.1 then it may be removed from validation results and the test repeated using a fresh aliqout. Blakemore *et al* 2010. found that when the SPC internal control Ct result is above 34.0 considerable variation (increase) in Ct and detection results were noted.
2. Unexpected results When an unexpected result is encountered (unless the expected results is "MTB Not Detected"), add an additional 2 ml of SR to the remaining original sample and re-run within 4 hours of original sample preparation. If the retested result matches the expected result, replace the unexpected result with the retested result.
3. The sample fails validation if the retested result matched the original unexpected result.The sample fails validation if repeat testing is not possible, or more than 4 hours has elapsed since original sample preparation, or there is no way to determine if the unexpected results were due to sample or cartridge failure.
4. The sample fails validation if MTB is detected when the expected result is "MTB not Detected" for one or more aliquots.
5. Select another stock sample to replace the failed sample, dilute, aliquot, and validate as described above.

6.5.3 Labelling and Packaging of PT items

See SOP PT 013 Packaging, labelling and distribution of PT items

6.5.4 Homogeneity and Stability

(a) Homogeneity

The laboratory shall ensure the preparation procedure above is strictly followed to ensure homogeneity

Procedure for ensuring homogeneity

- Potential Isolates/strains are stored at -80°C to minimise genetic changes
- Number of subcultures is limited to 3 -6 times
- Isolates are selected from strains that had good concordance (at least 80%) in the SRLN rounds
- Sub culture of isolates/strains on LJ culture and selecting those showing those with easiest growing organsims
- Use of sterile new beads allows breakdown of colonies to get a homogenous suspension
- Preparation of McFarland 1.0 standard from the postive LJ slant and use of cell culture flasks
- Several sterility checks in the procedure above allow to discard any strain contaminated

Measurement of homogeneity

1. The QC/validation tests done above are taken as the measure of homogeneity.
2. For homogeneity to pass the % CV is $\leq$ 10, SD $\leq$ 3 and a mean of between (16-23) performances must be obtained.

(b) Stability

Procedure for ensuring stability during transportation

- Use of IATA triple packaging ensures no breakage of materials

Measurement of stability

The PT panel to be tested for stability must mimic the transport conditions of the dispatched panels as much as possible i.e. packaged and left at room temperature

One PT panel retained at NTRL is tested at least two weeks by doing GeneXpert MTB/RIF® analysis.

6.5.5 Storage and transport

See SOP PT 012 Handling and Storage of PT items.

6.5.6 Retaining of PT items

- Prepare and retain at least twenty (20) complete PT sets for each round
- Follow steps below Section 6.7

6.7 Retaining PT items

- PT items (reserve PTs) are retained for purposes such as (i) for sending any participating lab in case of PT loss or damage etc (ii) to test for stability at the end of the testing period (iii) trouble shooting (iv) training purposes among others.
- All PT items retained or reserve PT items are labelled in the same way as the PT items dispatched to participants as described in *See SOP PT 013 Packaging, labelling and distribution of PT items* with PT ID, round number and Year and put in either slide box(es) or cryo boxes.
- Store the retained PT items at appropriate conditions i.e.
    - **Microscopy PT**: ambient temperature in the microscopy training laboratory in the locker designated for Microscopy PT preferably for not more than 6 months.
